# Management of Maxillary Sinus Inverted Papilloma Using Endoscopic Modified Medial Maxillectomy (EMMM) and Direct Approach to the Anterior and Lateral Maxillary Sinus (DALMA) With Nasolacrimal Duct Preservation: A Case Report

**DOI:** 10.7759/cureus.112755

**Published:** 2026-07-15

**Authors:** Sreynith Keo, Vanthan Srey, Sophea Siv

**Affiliations:** 1 Otolaryngology - Head and Neck Surgery, Preah Ang Duong Hospital, Phnom Penh, KHM

**Keywords:** cerebrospinal fluid leak, en bloc resection, inverted papilloma, malignant transformation, minimally invasive technique, modified medial maxillectomy, nasolacrimal duct preservation, orbital injury, recurrence, synechia

## Abstract

Inverted papilloma is a benign sinonasal tumor characterized by local aggressiveness, a high recurrence rate, and potential malignant transformation. This report presents a case of inverted papilloma originating in the maxillary sinus, successfully treated using a fully endoscopic surgical approach with en bloc resection. This case demonstrates the safety and efficacy of endoscopic techniques for managing maxillary sinus inverted papilloma while preserving the nasolacrimal duct; it also highlights the importance of meticulous surgical planning, intraoperative precision, and long-term follow-up to optimize outcomes and reduce recurrence risk. Postoperative recovery was uneventful, and the 12-month follow-up demonstrated no evidence of recurrence or complications.

## Introduction

Inverted papillomas are unique sinonasal neoplasms that arise exclusively from the Schneiderian mucosa, a specialized ectodermally derived respiratory epithelium lining the nasal cavity and paranasal sinuses. Anatomically, the lateral nasal wall, specifically in proximity to the middle turbinate and the uncinate process, represents the most frequent site of origin, followed directly by primary development within the maxillary and ethmoid sinuses. When evaluating common patterns of local extension, the maxillary sinus is the most frequently involved paranasal cavity, with tumors often originating from or extending through the medial wall or sinus ostium. Secondary extension into the ethmoid sinus is likewise highly prevalent, typically occurring in direct conjunction with primary lateral nasal wall disease [1].

Epidemiologically, inverted papilloma accounts for 0.5-4% of all primary sinonasal tumors, with an annual incidence of approximately 0.5-1.5 cases per 100,000 population [2]. The disease typically presents at an average age of 53-55 years, with a classic male-to-female ratio ranging between 3:1 and 5:1 [3]. 

Endoscopic approaches provide direct visualization of tumors, allowing for precise excision while preserving surrounding structures and minimizing surgical morbidity. This report presents the surgical techniques employed and the outcomes, along with a discussion of the benefits, challenges, and implications of endoscopic resection in the treatment of sinonasal inverted papilloma while preserving critical anatomical structures (specifically the nasolacrimal duct). However, addressing lesions in the anterior and lateral maxillary sinus remains a technical challenge due to their location. This case report presents a combination of septoplasty, endoscopic sinus surgery (ESS) [4,5], transseptal access with crossing multiple incisions (TACMI) [6,7], endoscopic modified medial maxillectomy (EMMM) [8-13], and a direct approach to the anterior and lateral maxillary sinus (DALMA) [14,15].

## Case presentation

A 38-year-old female patient, a housewife with an unremarkable medical and family history, presented with a two-year history of unilateral right nasal blockage, facial pressure, and recurrent epistaxis. She was initially treated by a primary care provider for chronic rhinosinusitis with nasal steroid spray and oral antibiotics, which provided no symptomatic relief. 

**Figure 1:**
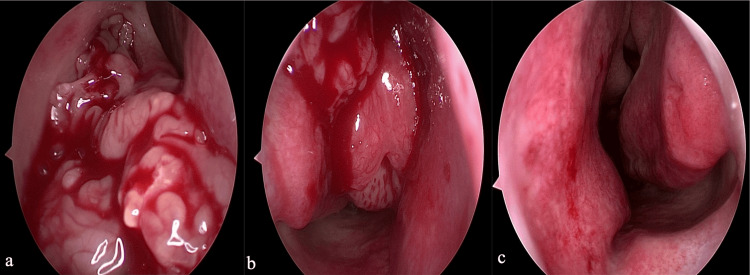
Preoperative endoscopic view (a) Right nasal cavity showing the lobulated tumor completely massed within the nasal cavity; (b) Right nasal cavity demonstrating a polypoid mass extending from the lateral wall of the nasal cavity; (c) Left nasal cavity showing no evidence of tumor extension.

An anterior rhinoscopic examination revealed a near-total occlusion of the right nasal passage by a solitary and lobulated mass. Endoscopy revealed the expansile soft tissue mass with variable consistency, a pink appearance, and a tendency to bleed. That tumor occupied the entire right nasal cavity and extended into the right maxillary sinus (Figure 1). 

**Figure 2:**
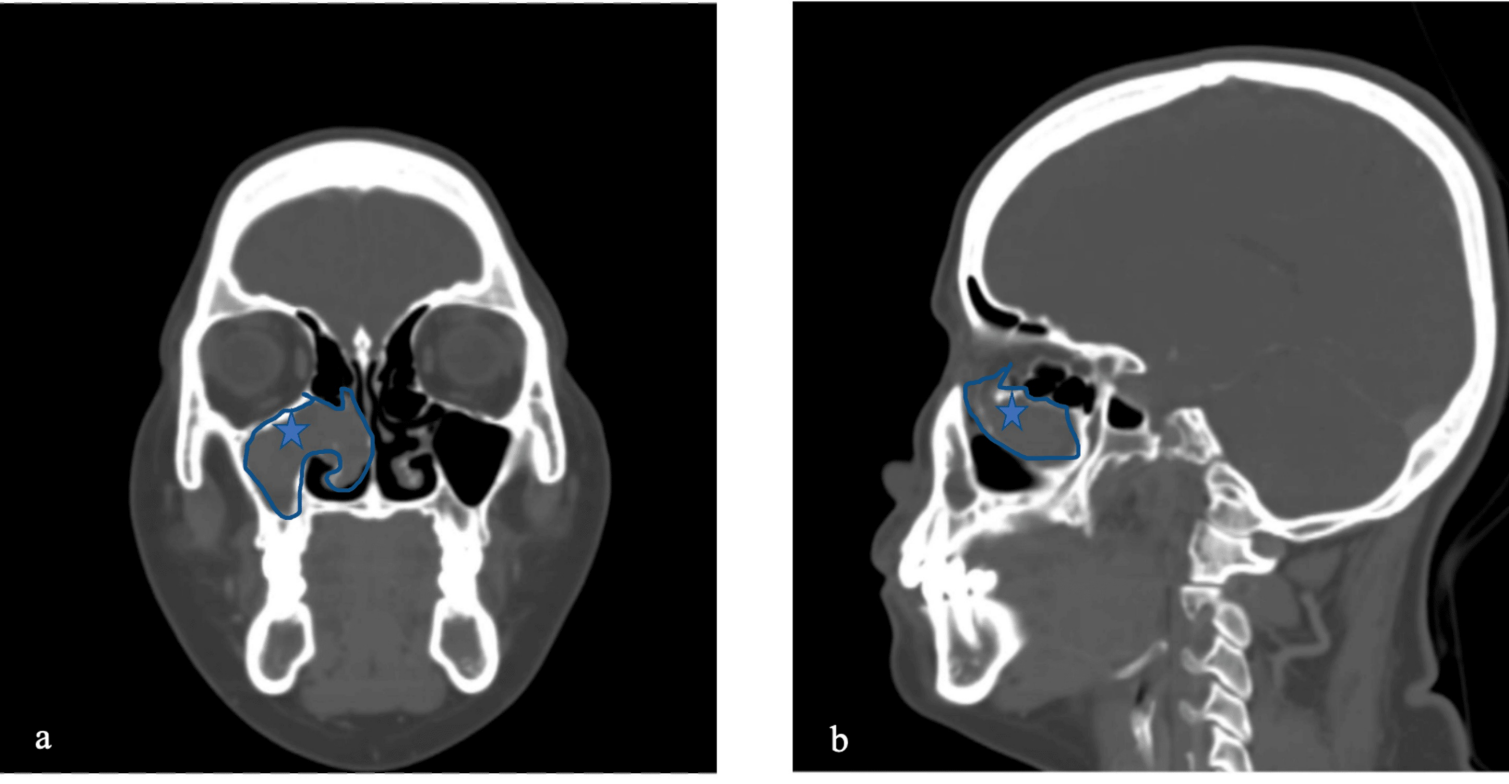
Preoperative contrast-enhanced computed tomography (CT) scan of the paranasal sinuses. (a) coronal view (bone window), and (b) sagittal view (bone window). The blue star indicates the tumor's pedicle of inverted papilloma.

Unenhanced paranasal computed tomography (CT) in the bone window revealed a right nasal fossa and maxillary sinus tumor measuring 38x35x44 mm that demonstrated enhancement following contrast injection. The bone appeared thickened and dense, presenting as a sclerotic spur at the superomedial wall of the right maxillary sinus and extending into the anterior ethmoid. Bony thinning and remodeling were visible at the floor of the eye socket, compressing the adjacent soft tissues within the lower orbit. Furthermore, the massive sinonasal tumor demonstrated pterygopalatine fossa involvement characterized by significant displacement, though notably without direct tissue invasion or infiltration (Figure 2).

Based on this radiological evidence of extensive tumor attachment at the superomedial wall and extension abutting the anterior and posterior dimensions of the maxillary sinus, the disease was classified as Stage T3 according to the Krouse staging system [16,17] (defined by tumor involvement extending beyond the isolated medial wall to other sinus boundaries), which correspondingly correlates to Group B within the Cannady classification [18]. 

Histopathology confirmed a diagnosis of sinonasal papilloma, inverted type. Endoscopic endonasal surgery was performed to remove the nasal tumor and relieve the patient's primary symptoms of obstruction and facial pain.

Surgical procedure

Preoperative preparation included administration of antibiotics and nasal decongestants. The surgery was performed using a purely endoscopic approach, with the patient under general anesthesia. Septoplasty was performed to correct the septal deviation by resecting the deviated cartilage and bone. A vertical incision was made on the left side, based on the Killian method. TACMI was performed using vertical and horizontal septal incisions on the right side to improve bilateral nasal access and tumor mobilization. 

**Figure 3:**
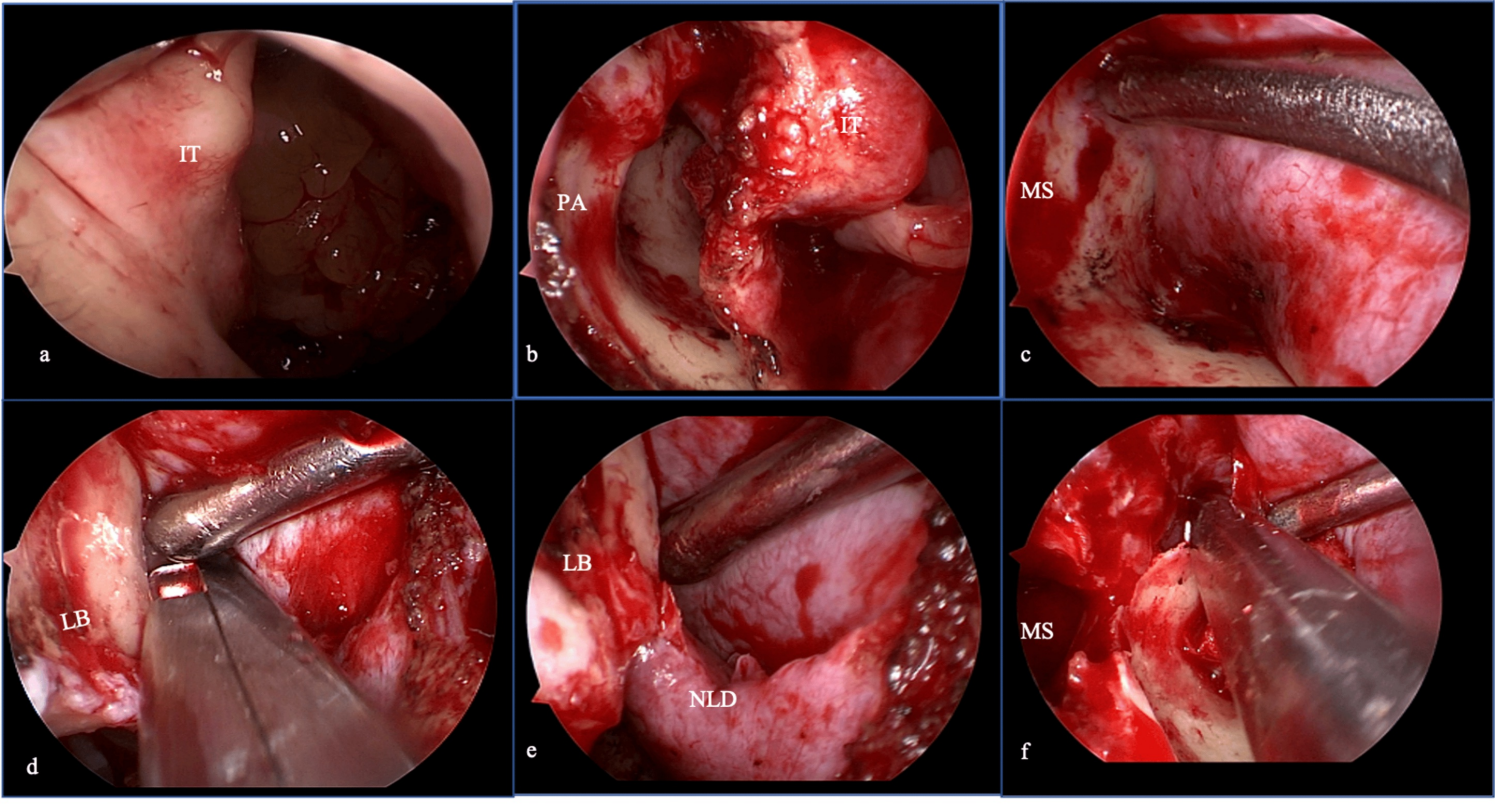
Intraoperative endoscopic view of EMMM (a-c): Initial exposure and access to the interior of the maxillary sinus; (d-f): Visualization and lacrimal bone removal executed to reveal the superior part of the naso-lacrimal duct and maxillary sinus PA: piriform aperture; IT: inferior turbinate; MS: maxillary sinus; LB: lacrimal bone; NLD: naso-lacrimal duct; EMMM: endoscopic modified medial maxillectomy

EMMM was performed. A mucosal incision was made along the piriform aperture to establish the initial surgical entry point. The lacrimal process of the inferior turbinate, the frontal process of the maxilla, and part of the lacrimal bone were drilled to expose the superior portion of the lacrimal duct. The interior of the maxillary sinus was visualized from the inferior meatus side. The inverted papilloma was observed on the anterior inferolateral wall, allowing for targeted interventions such as localization, pedicle attachment mapping, and successful resection (Figure 3). 

While EMMM provided excellent inferior meatal visualization of the maxillary sinus, utilizing the DALMA approach further enhanced surgical access, specifically to the anteroinferior and anterolateral corners of the sinus cavity. The mucosa of the antero-lateral nasal wall was elevated at the incision site near the piriform aperture. Submucosa dissection was extended laterally to identify the critical landmark.

**Figure 4:**
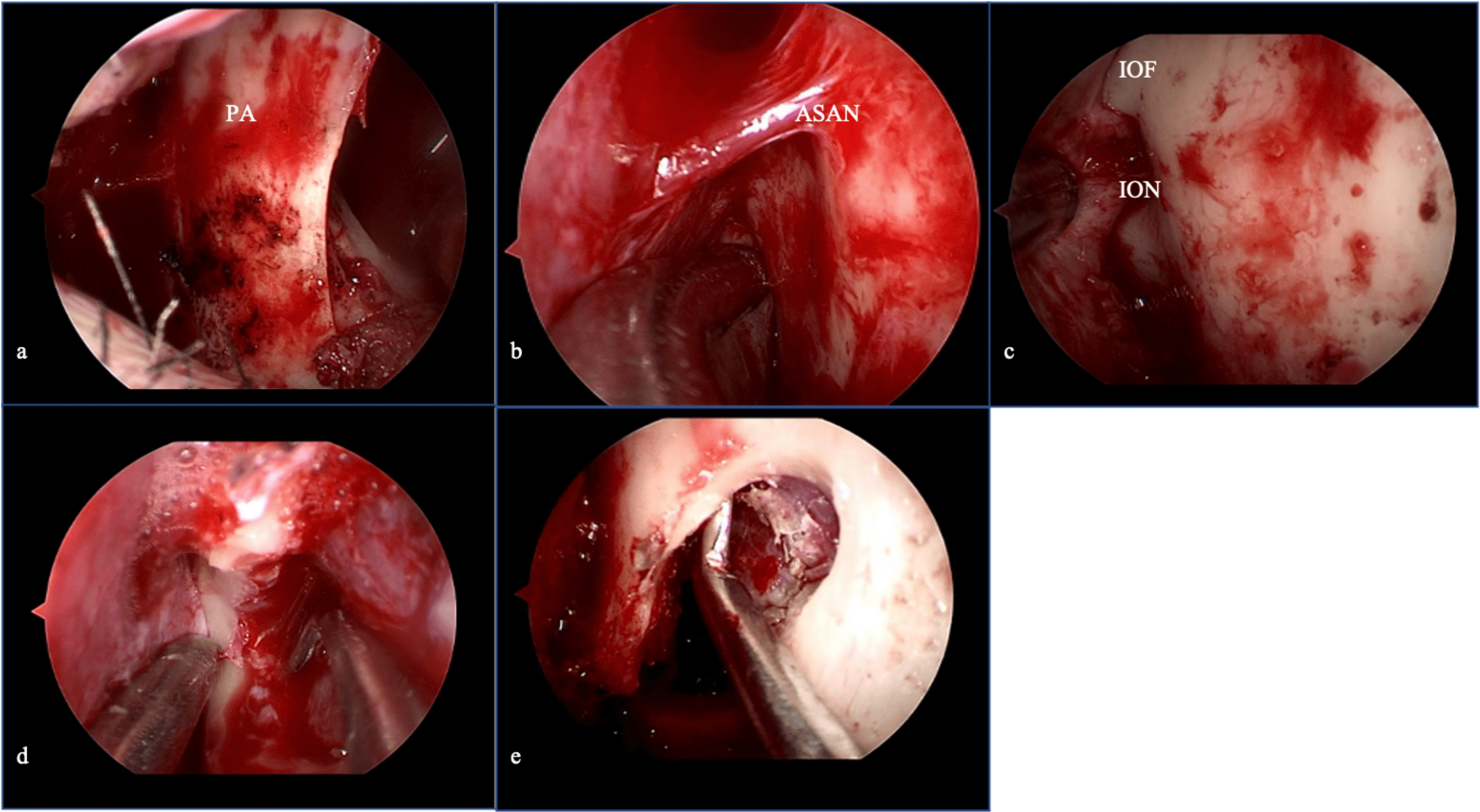
Intraoperative endoscopic view of DALMA (a–c): elevation of the antero-lateral nasal wall mucosa at the incision site near the PA, followed by identification of the ION, IOF, and the ASAN; (d–e): resection of the PA to achieve an enhanced surgical view of the lateral wall of the maxillary sinus. PA: piriform aperture; ASAN: anterior superior alveolar nerve; IOF: infraorbital foramen; ION: infraorbital nerve; DALMA: direct approach to the anterior and lateral maxillary sinus

The infraorbital foramen was carefully exposed, allowing direct visualization and protection of the infraorbital nerve (ION) and the anterosuperior alveolar nerve (ASAN) to prevent postoperative numbness. Once the vital nerves were mapped and safely shielded, the bony piriform aperture was drilled through the anterior face of the maxilla. Removing this specific bone boundary drastically improved the instrument's maneuverability and structural visibility within the sinus cavity. This created an unhindered line of sight directly into the anteroinferior and anterolateral corners of the maxillary sinus, giving full access to resect IP with clean margins (Figure 4).

**Figure 5:**
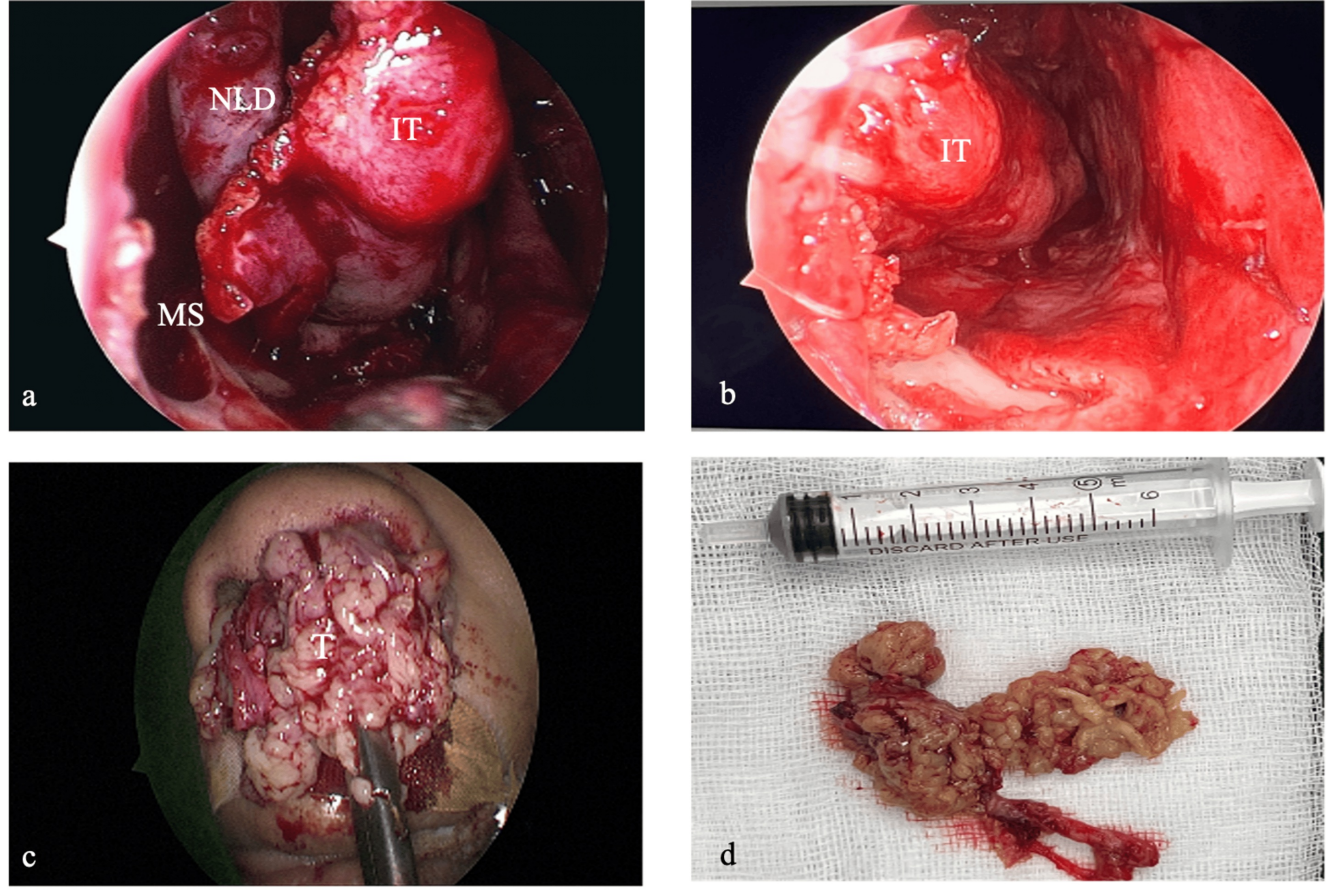
Intraoperative view demonstrating en bloc tumor resection (a): Endoscopic view demonstrating the regional anatomy, highlighting the nasolacrimal duct and the inferior turbinate; (b): Visualization of the surgical cavity following successful tumor clearance, with reconstruction of the nasal mucosa by suturing; (c):  Inverted papilloma view being isolated and extracted; (d): A gross specimen of the completely excised tumor lay alongside a syringe for size reference before histopathological analysis. MS: maxillary sinus; IT: inferior turbinate; NLD: nasolacrimal duct; T: tumor

Simultaneously, a right-sided endoscopic sinus surgery was performed to identify the superior extent of the tumor. The attachment of the tumor at the supero-medial wall of the maxillary sinus was identified and completely resected by drilling the hyperostosis to minimize the likelihood of recurrence. Finally, the sinonasal mucosa was reconstructed by suturing. The entire specimen was sent for histopathology to confirm the absence of malignancy (Figure 5). Hemostasis was achieved using bipolar cautery, SURGICEL™ (Johnson & Johnson, New Brunswick, New Jersey, United States), and nasal packing.

Postoperative care

**Figure 6:**
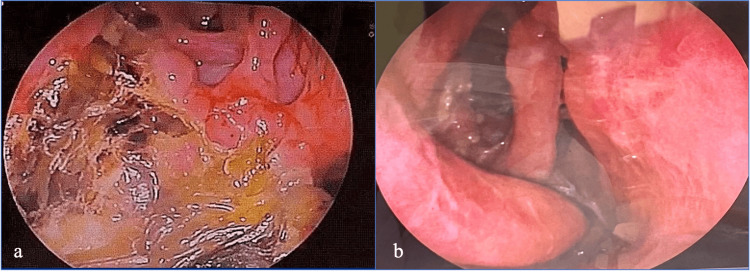
Postoperative endoscopic view: (a) Six-month and (b) 12-month postoperative follow-up demonstrates a well-healed surgical cavity without evidence of recurrence and complications.

Postoperative care included nasal irrigation, decongestants, antibiotics, pain control, and regular endoscopic cleaning to prevent crusting and synechiae formation. Weekly endoscopic debridement was performed initially. No complications, such as significant bleeding, orbital injury, or cerebrospinal fluid leak, were observed. The patient recovered uneventfully, with no evidence of recurrence or residual disease at the 12-month follow-up (Figure 6). Nasal obstruction and epistaxis resolved completely. Histopathology confirmed an inverted papilloma with clear surgical margins and no evidence of malignant transformation.

## Discussion

Endoscopic techniques have revolutionized the management of sinonasal tumors, providing significant advantages over traditional open surgical approaches. In the management of inverted papilloma, endoscopic resection offers reduced patient morbidity, superior visualization of the intricate nasal anatomy, significantly shorter recovery times, and excellent cosmetic outcomes. Furthermore, the literature demonstrates that these minimally invasive techniques achieve comparable or lower recurrence rates while crucially preserving nasal physiology and functional anatomy [4,5]. In the present case, utilizing a purely endoscopic approach culminated in complete tumor removal with no reported complications and a disease-free status at the 12-month follow-up mark.

However, managing an inverted papilloma that originates within the maxillary sinus presents unique anatomical hurdles. Recent literature emphasizes the necessity of balancing general endoscopic benefits against the specific technical challenges posed by the maxillary sinus wall, particularly regarding difficult tumor attachment sites and their subsequent impacts on long-term patient quality-of-life outcomes [2,3,6]. Historically, accessing the far anterior and lateral walls of the maxillary sinus required open procedures like the Caldwell-Luc approach [19,20], which carried a higher risk of facial numbness, prolonged swelling, and chronic pain. While the extended endoscopic Denker’s approach was subsequently developed to overcome these visualization limits by sacrificing the piriform aperture, it still carries an elevated risk of permanent upper lip numbness, alar notching, and structural alterations to the nasal valve area [21]. By contrast, adopting a more tailored endoscopic strategy in this case successfully circumvented both the morbidity of external incisions and the structural destabilization associated with extensive anterior bone removal, while maintaining the wide-angled visualization required to safely clear the sinus boundaries.

To achieve complete tumor clearance without resorting to highly invasive open or radical endonasal resections, advanced endoscopic strategies are now frequently utilized. Endoscopic resection, specifically incorporating EMMM and direct approaches to the anterior and lateral maxillary sinus, has emerged as a safe, highly effective alternative to the traditional Denker's procedure for managing IP originating within these difficult subsites [1,7,8,22-26]. While Denker's approach offers uncompromised lateral reach, EMMM provides a more conservative pathway that routinely preserves the nasolacrimal duct and the functional architecture of the inferior turbinate, thereby dramatically reducing postoperative epiphora and crusting. The successful 12-month recurrence-free outcome observed in our patient strongly aligns with the clinical data established by these larger cohort studies. By utilizing EMMM, we obtained the precise surgical angles necessary to meticulously drill the tumor attachment site, minimizing the risk of recurrence while optimally preserving long-term sinonasal function.

## Conclusions

Endoscopic resection is a safe and effective method for treating inverted papilloma of the maxillary sinus. Careful preoperative planning, meticulous surgical technique, and long-term follow-up are critical to optimizing outcomes and minimizing recurrence risk. Early diagnosis, precise imaging, and complete resection of tumor attachment remain the cornerstones of successful treatment and prevent recurrence.
